# Immunogenicity of MHC Class I Peptides Derived from *Leishmania mexicana* Gp63 in HLA-A2.1 Transgenic (HHDII) and BALB/C Mouse Models

**Published:** 2012

**Authors:** H Rezvan, R Rees, SA Ali

**Affiliations:** 1 Dept. of Laboratory Science, School of Paraveterinary Sciences, Bu-Ali Sina University, Hamadan, Iran; 2 School of Science and Technology, Nottingham Trent University, Clifton, Nottingham, UK, NG11 8NS

**Keywords:** HHDII, Peptide, Vaccine, *Leishmania*

## Abstract

**Background:**

*Leishmania* is an intracellular parasite infecting humans and many wild and domestic animals. Recent studies have suggested an important role for cytotoxic T cells against *Leishmania*. Peptide-based vaccines targeting short sequences derived from known immunogenic proteins have been shown to elicit cellular immune responses against disparate pathogens.

**Methods:**

We predicted four HLA-A2 peptides derived from *L. mexican/major* gp63 and tested these in HHD II mice, as well as four peptides for mouse MHC class I from the same proteins tested in BALB/ mice.

**Results:**

The results revealed immunogenicity for three of the four peptides predicted for HLA-A2. Immunisation with these peptides, along with IFA, induced CTL responses detected by standard 4-hour cytotoxicity assay and significantly upregulated the production of IFN-γ. When HHDII mice were injected IM with *L. mexicana* gp63 cDNA and splenocytes were restimulated with blasts loaded with the immunogenic peptides, two of the peptides were able to induce significant level of IFN-γ detected by ELISA. None of the peptides predicted for Balb/c mouse MHC class I elicited CTL activity or significantly upregulated the IFN-γ.

**Conclusion:**

The results may help in developing a peptide-based vaccine, which can be applied alone or in combination with drugs against *Leishmania*.

## Introduction

Leishmaniasis is a worldwide human and animal disease caused by malaria-like parasites called *Leishmania*. Today, leishmaniasis is considered endemic in 16 developed and 72 developing countries. An estimated 12 million human cases of leishmaniasis exist worldwide and a further 367 million is at the risk of acquiring the disease. Also, an estimated number of 1.5-2 million new cases are occurring annually (1–4). Human leishmaniasis and HIV virus co-infection, especially among adults, has also been progressively increasing as HIV-positives are now accounted for about 50% of all adult visceral leishmaniasis cases (1, 5). In mammalian hosts, there is convincing evidence to suggest that the immunity against *Leishmania* parasites relies on generating a cellular immune response (Th1). This is clearly demonstrated by the genetic predisposition of susceptibility to *L. major* infection in BALB/c mice, which correlates with the domination of IL-4-driven Th2 response and resistance in C57 ones, linked to an IL-12-driven, interferon-γ (IFN-γ)-dominated Th1 response that promotes parasite clearance in this model (6).

In *Leishmania*, the function of CD8+ T cells in generating immunity to the parasite was undefined for many years. However, the role of these cells in pathogenesis and immunity to *Leishmania* was confirmed later where immune BALB/c mice rechallenged with *L. major* showed production of IFN-γ from CD8+ T cells (7). CD8+ T lymphocytes are highly cytolytic in vitro against *Leishmania*-infected macrophages (8). So, it is now believed that CD8+ T cells take a significant part in immunity against *Leishmania*.

Different strategies have been considered to develop an effective vaccine for leishmaniasis but according to WHO's report, there is not yet wholly effective vaccine available for that, although different preventive or even therapeutic vaccines are currently under investigation (9, 10). Gp63 is an extremely potent immunogenic protein. Injection of this protein in saline together with Complete Freud's Adjutant (CFA), BCG and *Corynebacterium parvum* induced significant protection in BCA mice, where the levels of protection was dependent upon the site of vaccination relative to that of the challenge infection (11). Our studies have also shown high levels of immunogenicity for *Leishmania* gp63 DNA particularly using gene gun (12). In addition, proteins taken up by antigen presenting cells (APC) are processed into short peptides and presented to T cells through MHC class I and II molecules.

The aim of the study was based on this fact that identification of strong immunogenic peptides derived from *Leishmania* gp63, which have high affinity to MHC class I or II and are presented by APCs would be a feasible strategy for developing a novel vaccine against *Leishmania* parasites.

## Materials and Methods

### Cells & Animals

The CT26 cell line (N-methylurethane-induced BALB/c murine colon carcinoma) was a kind gift from Prof. Ian Hart (St Thomas Hospital). Cells were cultured and maintained in Dulbecco's modified Eagle's medium (DMEM) + 10% foetal calf serum (FCS) (Bio Whittaker, Europe).

HLA-A0201 transgenic (HHDII) mice, a generous gift from Dr. F Lemonnier **(**Institute Pasteur, Paris) were housed and bred at the Nottingham Trent University (NTU).

BALB/c mice were purchased from the Harlan Olac (Oxon, UK) housed and bred at the Nottingham Trent University. All animals were handled in accordance with the Home Office Codes of Practice and the NTU Ethical Review Committee for the housing and care of animals.

### Leishmania parasites

*Leishmania mexicana* strain M379 was obtained from Dr V. Yardley, the London School of Hygiene and Tropical Medicine (LSHTM), and routinely cultured in Schneider media (Sigma, Missouri, USA), supplemented with 10% FCS at 25 °C as described by Bates (13).

### Peptides

A list of peptides used in this study is shown in the Table 1. The peptides were predicted by SYFPETHI web-based software and synthesised by Alta Bioscience. Peptides were selected based on having high prediction score on SYFPETHI software and being shared among more *Leishmania* species.

**Table 1:** *L. mexicana/major* gp63 peptides predicted mouse/human MHC Class I a peptide prediction web-based software (SYFPETHI)

Gene	Sequence	Abbreviation	Type of MHC Class I	Score of peptides in SYFPEITHI
*L. major* gp63	LLVAALLAV	B8	HLA-A2	28
*L. mexicana/ major* gp63	RLAAAGAAV	C2	HLA-A2	25
*L. major* gp63	RLSLGACGV	C1	HLA-A2	23
*L. mexicana/major* gp63	AAAGAAVTV	CM4	HLA-A2	24
*L. mexicana/major* gp63	YYTALTMAI	A3	H2-Kd	21
*L. mexicana/major* gp63	DYTNCTPGL	A4	H2-Kd	20
*L. mexicana/major* gp63	VPNVRGKNF	A5	H2-Ld	22
*L. mexicana/major* gp63	ASLLPFNVF	A6	H2-Ld	21

### Immunization to test CTL activity of immunogenic peptides

Immunization was undertaken as previously described by Ahmad (14). Briefly the procedure was as follows:

*HHD II mice:* 100µg of the peptide, 140µg of HAP-B as helper peptide and 50µl IFA were transferred to a 1.5ml epindrof tube. PBS was added in a total volume of 100µl per mouse. The injection was given at the base of the tail. Mice were euthanized one week after the immunisation and their splenocytes were used in standard 4-hour cytotoxicity assay. *BALB/c mice:* Peptides were prepared similar to those of HHDII mice with the exception of using a 15 mer peptide derived from bovine albumin with the sequence of ISQAVHAAH AEINEAGR as the helper peptide. Mice were injected twice two weeks apart at the base of the tail and one week after the second immunization they were euthanized and their splenocytes were tested for CTL activity.

### Coating of gold particles by DNA

DNA was coated onto 1µm gold particles (Biorad, Hemel Hempstead, Hertfordshire, UK) as per manufacturer's instructions and administered by Helios Gene Gun (Biorad). Briefly, 200µL of spermidine was added to 16.6µg of gold followed by sonication; 200µl of 1 M calcium chloride was added to the DNA–spermidine solution followed by incubation at room temperature for 10 min. Tubes were centrifuged at 20780 g for 1 min and the gold particles resuspended in dry ethanol (Sigma). After repeating the above step twice, particles were resuspended in 0.025 mg/ml of polyvinylpyrrollidone (PVP) in dry ethanol. During these steps, the gene gun plastic tube was dried for 15–20 min using nitrogen gas. The resuspended gold particles were loaded onto the dried tube using a syringe and the tube was placed on the roller / dryer (Biorad) followed by incubation for 15 min. The PVP–dry ethanol was gently removed using the syringe and the tube was rotated on the roller along with nitrogen gas being passed through it for 5 min. Bullets were then cut to a measured size using a guillotine and stored at 4°C until used for immunization.

### Gene gun immunization with Leishmania mexicana gp63 cDNA

One microgram of *L. mexicana* gp63 cDNA plasmid construct (VR1012) coated on gold particles was administered to a shaved area of the abdominal skin of each BALB/c mouse by gene gun (Biorad) on days 0 and 14. The control group was given 1µg of the empty vector coated on gold particles or injected with PBS. On day 28 all mice were either euthanized for CTL assays or challenged with *Leishmania*, and were monitored regularly.

### I.M. Immunisation with L. mexicana gp63 cDNA

Two groups of 6 female BALB/c or HHDII mice were immunized I.M. with either 100µg/mouse *L. mexicana* gp63 plasmid DNA (VR1012 vector) or the empty vector as control in hind leg muscles. In some experiments, controls were injected with PBS. The mice were injected twice on day 0 and 14, and on day 28, they were either euthanized for CTL assays or challenged with *Leishmania* followed by regular monitoring.

### Animal infection with Leishmania parasite

Groups of 6 BALB/c and/or HHD II mice were routinely infected, unless otherwise indicated, by intradermal inoculation of 2×10^6^ and 1×10^7^ *L. mexicana* promastigotes in a volume of 50µl/mouse into a shaved area of the back region about 1 cm from the tail base respectively and were monitored at 3- to 4-day intervals. Mice were killed when lesion size exceeded 1 cm^2^.

### CTL Assay Preparation of LPS blast

In order to produce blast cells for in vitro restimulation, naive splenocytes were cultured for 3 days prior to the removal of spleens from immunized mice at 1.5×10^6^ cells /ml in 40-ml T-cell media (complete RPMI, 10% FCS (by volume), 1% glutamine (by volume), 20 mM HEPES, 50µM 2-mercaptoethanol, 50 U/ml penicillin /streptomycin, 0.25 µg/ml fungizone) containing 25µg /ml LPS and 7µg /ml dextran sulphate in T75 culture flasks incubated at 37 °C, 5% CO2. On day 2, cells were into two plates and pulsed with 10µg /ml of relative and irrelative peptides. LPS-treated naive splenocytes were irradiated at 3000 rads for 4 min. Cells were washed and pulsed again with 100µg /ml of the relevant and irrelevant peptides for at least 1 h. Cells were then washed, counted and added to culture plates containing splenocytes from immunized mice at 5×10^5^ per well.

### In vitro generation of CTLs

One week after the last immunization, spleens were harvested from immunized and control mice. Cells were flushed out from the spleens and processed as previously described (12). Splenocytes were plated in a 24-well plate at 2.5×10^6^ cells /500µl per well and 5×10^5^ /500µl irradiated and peptide-pulsed LPS blasts were added to make a final volume of 1 mL in each well of 24-well plates and incubated for 5 days at 37°C, 5% CO2. Supernatant was harvested for cytokine assay on day 2 and/or 5. On day 5 of in vitro stimulation, splenocytes were harvested, washed twice in serum-free medium, counted and resuspended in T-cell media and used as the effector cells. In certain experiments, CD8+ cells were depleted by using CD8 beads (Dynal, Wirral, UK) according to manufacturer's instructions. Target cells (RMA/S-A2 or T2 for HHD II mice & CT-26 or A20 for BALB/c mice) were also harvested, washed and labeled with 51chromium (Amersham, Buckinghamshire, UK) followed by 1 h of incubation at 37°C. The labeled target cells were then pulsed with relevant and irrelevant peptides separately (20µg/ml) and incubated for 1 h at 37°C. A standard 4-h 51Cr release assay was performed and the specific cytotoxicity was determined using the following formula.

$$\text{percentage cytotoxicity}=\frac{\left(\text{experimental release}-\text{spontaneous release}\right)}{\left(\text{maximum release}-\text{spontaneous release}\right)}\times 100$$

Maximum release: Chromium released from 51Cr labeled cells killed by 1% SDS and used as positive control

Spontaneous release: Chromium released from cells only labeled with 51Cr and used as negative control.

### Cytokine Assays (IFN-γ & IL-4)

Splenocytes were prepared as outlined above. 1 ml of the cell culture supernatant on days 2 and/or 5 was collected and stored at -20C until required. Cytokine analysis for IFN-γ and IL-4 using the ELISA kits (R&D Systems, Abingdon, UK) was performed according to the manufacturer's protocols.

## Results

### Immunogenicity of L. major/L. mexicana gp63 peptides predicted for HLA-A0201 in HHDII mice

Peptides from gp63 proteins of *L. major* and *L. mexicana* (gene bank ref X64394 and Y00647) were selected for HLA-A0201 class I molecules by using the prediction web-based software “SYFPEITHI” and their immunogenicity was determined in HLA-A0201 transgenic (HHDII) mice.

**Fig. 1:**
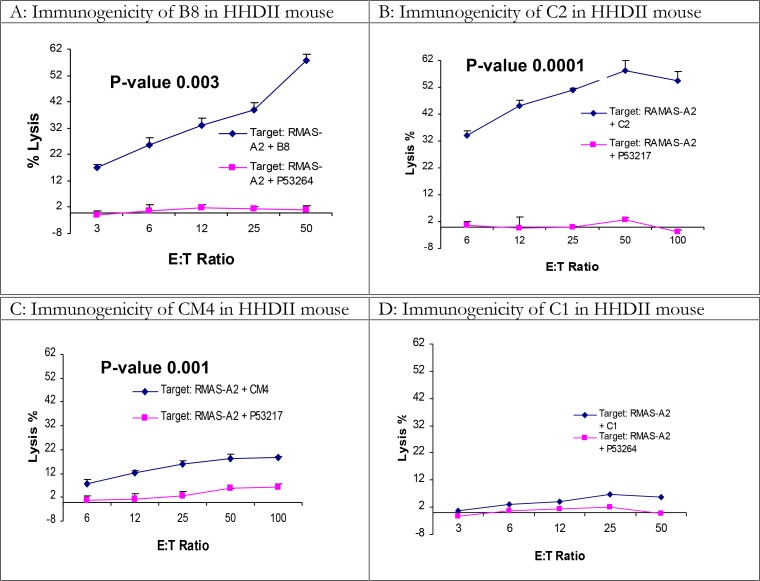
Immunogenicity of B8, C2 and C1 peptides in HHDII mice Four peptides of *Leishmania* gp63 proteins were predicted for HLA-A2.1 using SYFPEITHI web-based software. 100µg of each peptide was injected S.C. at the base of tail of HHDII mice together with the helper peptide and IFA adjuvant. A week after the immunization, spleens were harvested and splenocytes were cultured with spleen blast cells pulsed with relevant and irrelevant P53 peptides for 5 days. On day 5 the cells were used as effectors against target cells “RMAS-A2” pulsed with relevant and irrelevant P53 peptides using standard 4-hour cytotoxicity assay. Results of peptides B8, CM4 & C2 are representative of immunogenic peptides while peptide C1 represents a poor immunogenic peptide. Graphs represent a number of independent positive experiments as Table 3

Prior to the peptide immunization, the efficacy of the immunization and CTL experimental protocols was confirmed by immunization with the PAP135 peptide (sequence: ILLWQPIPV) as the positive control (12, 15). From *L. major/L. mexicana* gp63 four peptides, which are also shared in other *Leishmania* species (Table 2), were predicted for HLA-A0201 and their immunogenicity was tested in HHDII transgenic mice; a summary of the results are shown in Table 3 and Fig. 1. Mice were immunized once with 100µg of each peptide together with 100µg of IFA adjuvant and a helper peptide, which were administered S.C. at the base of the tail (see materials and methods). Two peptides (C2 and B8) were highly immunogenic and the immunogenicity of the third one (CM4) was less but, in comparison with the irrelevant peptide, still significant (*P*=0.001). The fourth peptide (C1) showed very weak immunogenicity. Boosting with a second immunization did not enhance the immunogenicity of the C1 peptide. *In vitro* depletion of CD8+ T cells inhibited the cytotoxicity indicating a role of CD8+ T cells as mediators of cytotoxicity (data not shown). Student *t*-test was performed to analyze all results.

**Table 2:** Presence of immunogenic peptides in *Leishmania* species

	*Leishmania* species						
Peptide code	*major*	*mexicana*	*donovani*	*infantum*	*aethiopica*	*chagasi*	*tropica*
B8	+	-	+	+	-	+	-
C2	+	+	+	+	+	+	+
CM4	+	+	-	+	+	-	+
C1	+	-	-	+	+	-	+

Data is generated from the alignment of different Leishmania species gp63 genes with the gene bank references of X64394, Y00647, P08148, A45621, B42049, A44951, PL0221, AAB96339, CAB06018, CAA68673, P08148, AAR32945, CAD58718, B42049, C42049, A44951, S19916, PL0221, CAD42818, CAD42817, CAD42816, CAD42815, CAD42814, CAD42813, CAD42812, CAD42811, CAD42811, AAC39120, AAB96339, CAA69349 and CAB06018

**Table 3:** Summary of the immunogenicity of gp63 HLA-A2 restricted peptides in HLA-A2.1 transgenic (HHDII) mice

	Peptide	Sequence	Gene	Mouse	Score of peptides in SYFPEITHI	Ratio of positive results to the number of cytotoxicity assays
1	C2	RLAAAGAAV	gp63	HHDII	25	4/5
2	CM4	AAAGAAVTV	gp63	HHDII	24	2/3
3	B8	LLVAALLAV	gp63	HHDII	28	5/5
4	C1	RLSLGACGV	gp63	HHDII	23	1/5

### Cytokine Production of Splenocytes of HHDII mice Immunised with gp63 peptides

**Fig. 2:**
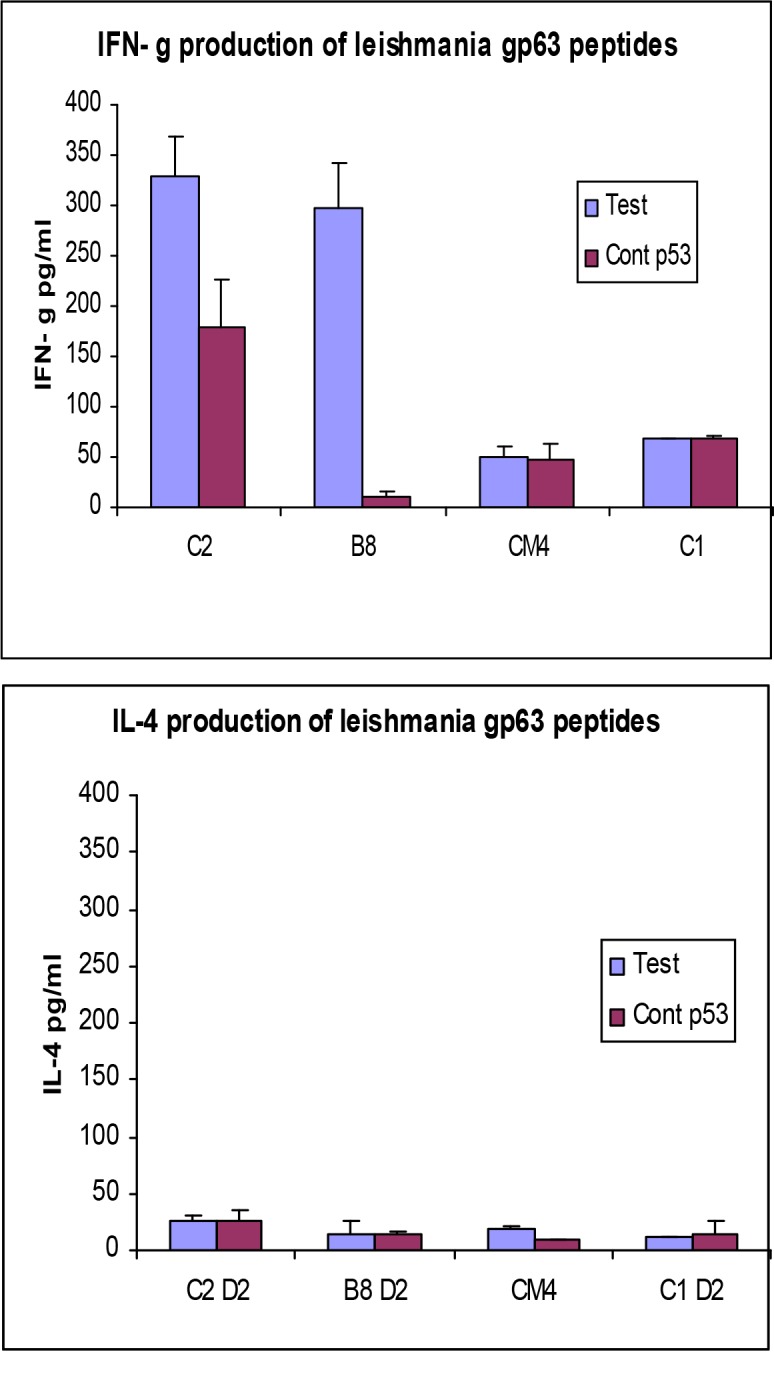
IFN-γ and IL-4 production by splenocytes cultured with relevant and irrelevant peptides. HHDII mice were immunized with the predicted peptides of gp63 and their splenocytes were cultured with splenocytes blast cells pulsed with the relevant peptides and an irrelevant peptide, P53”217” or PAP135, for 5 days. The supernatants were collected on day 2 and 5 and tested for IFN-γ and IL-4 using a commercial kit according to manufacturer's instruction. Student *t-*test was used to statistically analyze the results and P-value for the level of IFN-γ between test and control for peptides C2 and B8 was 0.015 and 0.009 respectively. Graphs represent a number of independent positive experiments as Table 3

In order to confirm the immunogenicity of C2, CM4, B8 and C1 peptides, the cytokine assays were conducted to detect IFN-γ and IL-4 on supernatants collected from immunized mouse splenocytes cultured for 2 days. The results are shown in Fig. 2.

For the highly immunogenic peptides (B8 & C2) the amount of IFN-γ detected in supernatants of splenocytes cultured with APCs pulsed with the relevant peptide was significantly higher than those cultured with blast cells (derived from mouse splenocytes) pulsed with the irrelevant peptide. For CM4 and C1, there was no significant difference in IFN-γ levels for splenocytes cultured with relevant peptides compared to those cultured with irrelevant ones. No significant IL-4 levels were detected for any of the peptides. Student *t*-test was performed to analyse the results (Fig. 2).

### Peptide vaccination in the BALB /c mouse model

**Fig. 3:**
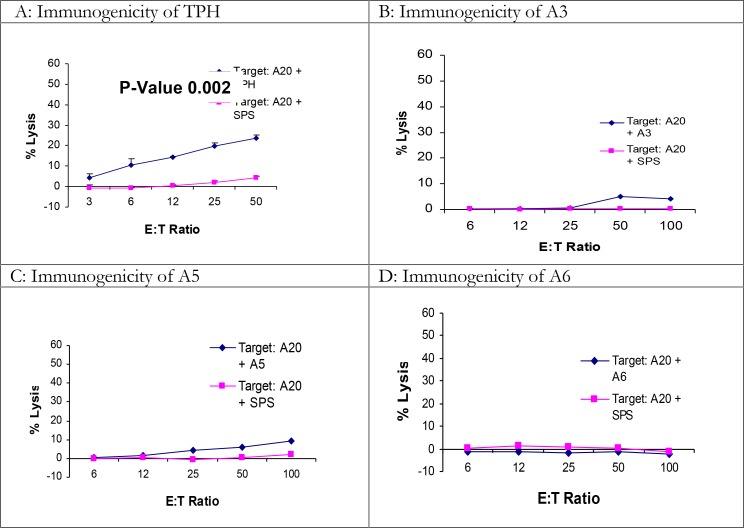
The immunogenicity of TPH and *L. major* gp63 peptides in BALB/c mice BALB/c mice were immunized twice at a week interval with 100µg of appropriate peptide together with the helper peptide and adjuvant (see materials and methods) S.C. at the base of the tail. A week after the last immunization, spleens were harvested and splenocytes were cultured with APCs pulsed with relevant and irrelevant “SPSYVYHQF” peptides for 5 days. On day 5 splenocytes were used as effectors in standard 4-hour cytotoxicity assay against targets pulsed with relevant and irrelevant peptides. Graphs represent a number of independent positive experiments as Table 3

To determine the efficacy of peptide vaccination in BALB/C mice, a 9 mere H2-L^d^ restricted peptide named TPH with the sequence of “TPHPARIGL” derived from β-galactosidase (12, 16), was used for immunization (see Materials and Methods). Also, four peptides derived from *L. major* gp63 protein, “A3, A4, A5 and A6”, predicted for MHC-class I H2-L^d^ and H2-K^d^ (Table 4) were assessed in BALB/c mice. Each mouse received two immunizations on days 0 and 7, and then the mice were euthanized for standard 4-hour cytotoxicity assay on day 14. The results clearly showed that immunization with some peptides induced low but significant levels of cytotoxicity against targets pulsed with the corresponding peptide using student *t*-test (Fig. 3). The frequency of positive results for TPH was quite low (Table 4).

**Table 4:** Evaluation of immunity induced by *L. mexicana* gp63 peptides predicted for mouse MHC class I using a web-based software (SYFPETHI) and tested in BALB/c mouse model

NO.	Peptide	Sequence	Gene	Mouse	Score In SYFPETHI software	Ratio of positive results to the number of cytotoxicity assays
1	TPH	TPHPARIGL	β-galactosidase	BALB/c	25	12/29
2	A3	YYTALTMAI	gp63	BALB/c	21	0/3
3	A4	DYTNCTPGL	gp63	BALB/c	20	0/4
4	A5	VPNVRGKNF	gp63	BALB/c	22	0/2
5	A6	ASLLPFNVF	gp63	BALB/c	21	0/4

Administration of mouse CpG or altering the time intervals of immunization failed to increase the immunogenicity of the predicted peptides and no significant increase of IFN-γ or IL-4 cytokines was observed when the splenocytes of immunized mice were cultured with blast cells pulsed with the relevant peptides (data not shown).

### Natural processing of the immunogenic class I peptides derived from Leishmania gp63

**Fig. 4:**
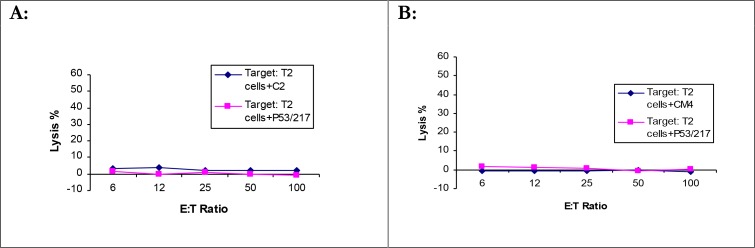
Assessment of natural processing of immunogenic peptides tested in HHD II mice HHD II mice were immunized by the gene gun with *L. major/L. mexicana* gp63 three times. After a week of the last immunization mice were killed and the CTL activity was determined by standard 4-hour cytotoxicity assay. Graphs represent 6 immunized mice for each peptide tested in three independent experiments. A: mouse immunized with C2 peptide B: mouse immunized with CM4 peptide. Graphs represent a number of independent positive experiments as Table 3

To evaluate the natural processing of the gp63 derived immunogenic peptides, DNA immunization by I.M. injection and gene gun were performed. Two plasmid constructs *L. mexicana* gp63 cDNA & *L. major* gp63 cDNA were used for immunization by the gene gun (HHD II & BALB/c). After 3 immunizations at 1 week interval, mice were killed and cytotoxicity assays were performed using tumor target cells pulsed with the relevant peptides as targets (RMAS for C2, CM4, B8 & C1 and A20 for A3, A4, A5 & A6) (see materials and methods). The results indicated that none of the peptides showed immunogenicity when mice immunized by gene gun using chromium release assay (Fig. 4).

The supernatant collected from the splenocyte cell culture were analyzed for IFN-γ and IL-4. No significant difference was observed between the level of IFN-γ or IL-4 in splenocyte cell culture supernatants cultured with APCs pulsed with relevant peptides (A3, A4, A5 & A6 for BALB/c and C2, CM4 & B8 for HHD II mice) and the irrelevant peptide (TPH for BALB/c & P53,264 for HHD II) by using student *t*-test (data not shown).

**Fig. 5:**
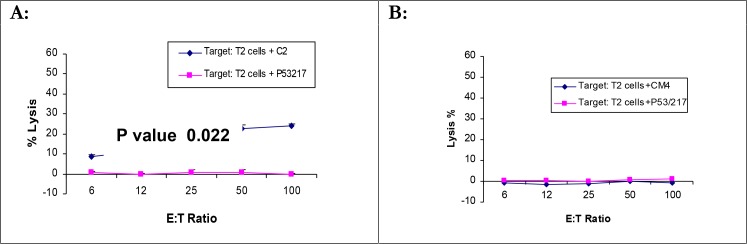
Immunogenicity of C2 and CM4 peptides by intramuscularly DNA immunisation BALB/c mice were intramuscularly injected with 100µg *L. mexicana* gp63 cDNA twice. Two weeks after the last immunization they were killed and the splenocytes were cultured with APCs pulsed with C2 and CM4 peptides for 5 days. On day 5, the splenocytes were used as effectors in standard 4-hour cytotoxicity assay against tumor cells pulsed with the relevant peptides. Only 1 out of 6 mice showed immunogenicity against targets pulsed with C2 peptide. A: mouse immunized with C2 peptide B: mouse immunized with CM4 peptide. Graphs represent a number of independent positive experiments as Table 3

The natural processing of C2 and CM4 (HLA-A0201 peptides/HHD II) was also assessed by intramuscular injection of DNA. Mice were injected IM with 100µg *L. mexicana* gp63 cDNA at two weeks interval. After two weeks of the last immunization, mice were euthanized and tested for the CTL activity as with that of gene gun immunization. The results showed CTL activity in 1/6 immunized mice detected by standard 4-hour cytotoxicity assay for C2 (Fig. 5).

**Fig. 6:**
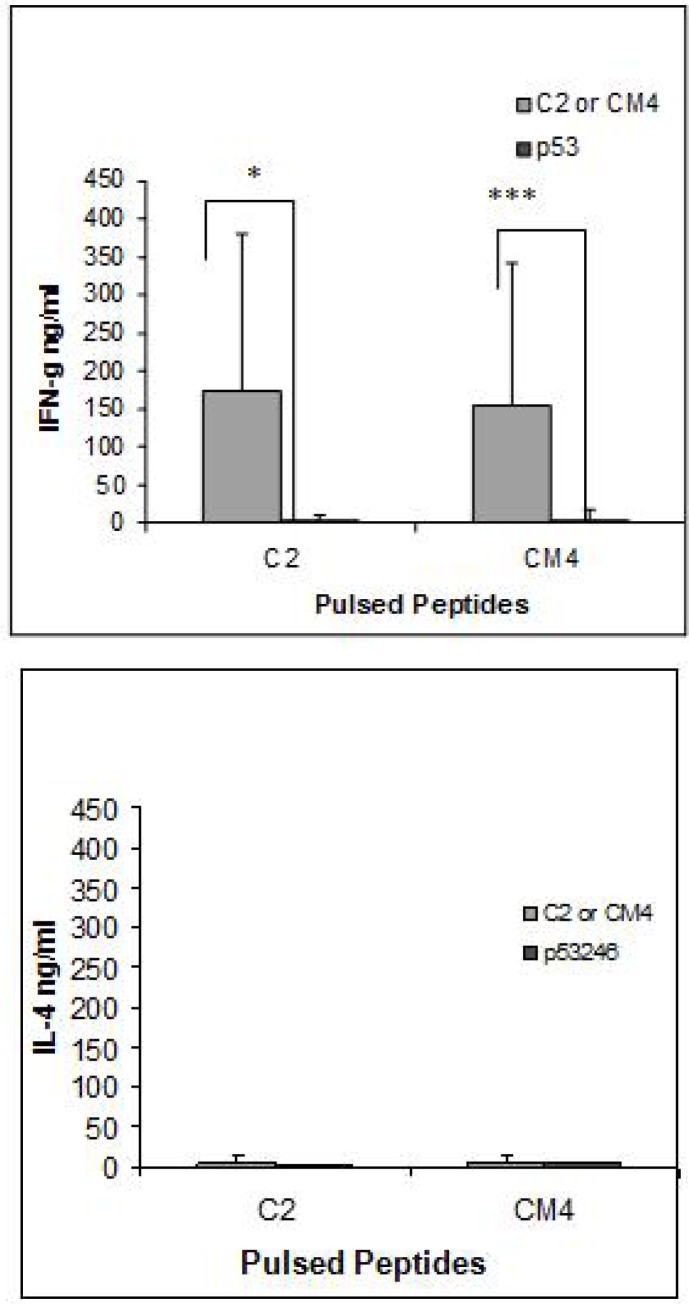
IFN-γ production of splenocytes from HHD II mice immunised with gp63 cDNA and stimulated with relevant and irrelevant peptides. Supernatants collected from the culture of the splenocytes were tested for IFN-γ by ELISA using the commercial kit according to the manufacturer's instructions. The graph represents three independent experiments and *P* value < 0.05, 0.01, 0.001 accounts for *, **, and *** respectively

The supernatants collected from i.m. immunized mice cultured splenocytes were tested for the presence of IFN-γ and IL-4. In contrast to the results of the cytotoxicity assay, there was a significant increase in the level of IFN-γ but not IL-4 in the supernatants of splenocytes obtained from the immunized mice when they were cultured with LPS blast cells pulsed with both C2 and CM4 peptides. Student *t*-test was performed to analyze the results (Fig. 6).

### Protection induced by immunisation with C2 peptide in HHDII mice

HHDII mice were immunized twice at two weeks intervals with 100 µg of the C2 and CM4 peptides. After two weeks of the last immunization, mice were challenged with 1×10^7^ log phase of *L. mexicana*. Mice were monitored for lesion development for at least 2 months. Two control groups of mice were used one injected with PBS and the other with an irrelevant peptide p53/246. The results showed no significant protection induced by immunisation with the peptides compared with controls using student *t*-test (data not shown).

## Discussion

### Peptide immunization in HHDII mice

Peptide immunization is a new vaccination approach that has been shown to be effective in inducing cellular and humoral immune responses. This new strategy has been implemented to test universal vaccine for certain pathogens like influenza or HIV based on the selection of conserved antigens that produce cross-protective humoral and cellular immunogenicity and the identification of immunogenic epitopes for T or B cells is the corner stone for such studies (17, 18). Gp63, a *Leishmania* antigen, has been postulated as a promising candidate for *Leishmania* peptide-subunit vaccine. In a study by Spitzer a 16-mer synthetic peptide with the sequence of YDQLVTRVVTHEMAHA derived from *L. major* gp63, induced a detectable immunity in BALB/c mice (19). On the other hand, there are many studies, including our own (12), which have demonstrated immunogenicity and CTL stimulation of *Leishmania* gp63 proteins suggesting a significant role for CD8+ T cells in immunity to *Leishmania*. Therefore, it is appropriate to identify the MHC calss I restricted CTL epitopes that can be used as a vaccine against *Leishmania* either alone or in combination with other immunogenic or therapeutic agents.

This study reports for the first time by using reverse immunology the identification of immunogenic MHC class I restricted epitopes from *Leishmania* gp63 protein in both HLA-A0201 transgenic (HHD II) and conventional BALB/c mouse models. In order to identify immunogenic epitopes, which are presented through MHC class I molecules, the web-based software “SYFPEITHI” (14, 20) was used to predict the immunogenic peptides for both models. The potency of SYFPEITHI software for prediction of *Leishmania* epitopes for HLA-A2 has been shown by Seyed (21).

The immunogenicity of the predicted peptides was determined by using a number of *in vivo* and *in vitro* immunological tests (17). Due to the ethical difficulties associated with studies on human subjects, HHDII mice were used to determine the immunogenicity of the peptides predicted for human HLA-A0201 molecules. HLA-A0201 transgenic (HHDII) mice have been described as a powerful model to study human immune responses *in vivo* (22–25). This mouse model has already been used to study *Tripanozoma cruzi* in the human (18). Although application of HHD II mouse model in *Leishmania* peptide vaccine studies is new, the results of this study was in line with those of other models (15). Using *L. major* gp63 sequences, four of nine mer peptides named C2, CM4, B8 and C1 (RLAAAGAAV, AAAGAAVTV, LLVAALLAV and RLSLGACGV) were predicted to have affinity to HLA-A0201 molecules and were tested for immunogenicity in HHD II transgenic mice. Three peptides (C2, B8 and CM4) induced CTL activity in the immunized mice; however, the CTL activity induced by CM4 was weaker. The fourth peptide (C1) was non-immunogenic and produced weak CTL activity. Injection of C2 and B8 together (the two high immunogenic peptides) failed to induce strong CTL activity against targets pulsed with either of the peptides in standard 4-hour cytotoxicity assay (data not shown) indicating the diversity of the immune response against two different immunogenic peptides. In order to obtain potent CTL activity the splenocytes were restimulated *in vitro* with APCs pulsed with the relevant peptide (see materials and methods). Either re-stimulation with no peptide or restimulation (with relevant peptides) without using APCs, failed to stimulate CTL activity indicating the importance of APCs in enhancing the CTL activity. Collectively data presented here and in other studies suggest that type of APC and antigen processing are crucial factors in CTL activation and must be taken in consideration in when synthetic peptides are selected.

IFN-γ secreted by T cells has been shown to be essential for the development of Th1 responses and it has been used as a marker for the existence of the CTL activity, while IL-4 on the other hand indicates the bias immunity towards the Th2 pathway (26). It has already been shown by us and other researchers that immunogenic peptides derived from tumour antigens can stimulate CTLs and IFN-γ production suggesting association with Th1 responses (27, 28). In addition, the level of IFN-γ produced by splenocytes from mice immunized with immunogenic *Leishmania* gp63 peptides (B8, CM4 & C2) cultured with APCs pulsed with relevant peptides confirmed its role in the activation of Th1 pathway and/or CTL responses. Immunization with the C1 peptide failed to produce a significant level of IFN-γ by using student t-test. The lack of IL-4 secretion may indicate down regulation or the absence of Th2 responses in this model. Our results were similar to those of other studies, which evaluated the immunogenicity of HLA-A2 restricted peptides derived from L. major using different models (21).

The potency of DNA vaccines in presenting particular epitopes has been already shown using epidermal immunization (29). To determine the natural processing of the immunogenic peptides, DNA immunization was performed using two methods; gene gun and intramuscular injection of the DNA, both were previously shown to induce protection against challenge with live parasites (12). *L. mexicana* gp63 cDNA construct was used to immunise for peptides C2 & CM4 and *L. major* gp63 cDNA for B8 and C1. Immunization of HHD II mice by the gene gun and restimulation the splenocytes with APCs pulsed with the corresponding immunogenic peptides failed to generate CTL activity, as measured by either standard 4-hour cytotoxicity assay or the production of IFN-γ in the splenocytes culture supernatants. In contrast, splenocytes from mice immunized by I.M. injection of cDNA restimulated with splenocyte LPS blasts pulsed with C2 or CM4 produced high levels of IFN-γ compare to those restimulated with APCs pulsed with an irrelevant peptide. In addition, a low frequency of CTL activity was detected by standard 4-hour cytotoxicity assay only for C2 peptide (1 out of 6 mice). The results indicate that these peptides may be naturally processed but the cytotoxicity assay is not sensitive enough to detect the immune responses, which are detectable by IFN-γ ELISA.

The potency of I.M. injection of DNA in inducing protection and CTL activity was in line with other studies, which reported CTL activity and protection induced by I.M. immunization of *Leishmania* gp63 and S.C. injection of ß-gal plasmid DNAs in BALB/c mouse model (12, 30) but BALB/c-based models are more potential than HHDII mice with C57 background, which are naturally resistant to some species of *Leishmania*, in demonstration of protection against *Leishmania* parasites.

### Peptide immunization in BALB/c mice

To develop a peptide-based vaccination model in BALB/c mice, which are sensitive to *Leishmania* parasites (22), an immunogenic 9-mer peptide “TPHPARIGL” derived from β-galactosidase protein and four peptides derived from gp63 with the high affinity to H2-Ld and H2-Kd (identified by SYFPEITHI software) were tested in BALB/c mice. Although TPH has shown strong immunogenicity when BALB/c mice were immunized with a Disabled Infectious Single Cycle Herpes Simplex Virus (DISC-HSV) virus encoding β-galactosidase protein followed by in vitro restimulation with TPH (16), immunisation with this peptide and adjuvant induced a low, but detectable, immune response (CTL activity) in 41% of immunised mice. Co-injection of DISC virus, CpG or Titer Max as adjuvants did not enhance the immunogenicity of the TPH peptide (data not shown). In addition, increasing the frequency of immunisation up to 3 times did not alter the immunogenicity of this peptide (data not shown). Increasing the frequency of in vitro restimulation (see Materials and Methods) did not positively alter the immunogenicity of peptides. When instead of the adjuvant IFA, Titer Max was used as an adjuvant and the splenocytes were pulsed by the relevant peptide without APCs according to the protocol used by Anne Saren (12), no killing of target cells pulsed with relevant peptide was observed compared to those pulsed with an irrelevant peptide (data not shown). Immunisation with peptides predicted for mouse MHC class I (A3, A4, A5 & A6 with the sequences of YYTALTMAI, DYTNCTPGL, VPNVRGKNF & ASLLPFNVF respectively) showed only low levels of immunogenicity indicating that the BALB/c system is a poor model for peptide vaccination in *Leishmania* possibly due to the bias of the immune system to a Th2 response rather than Th1 (6). The results were contradicted with the results obtained by Tabatabai when three peptidesderived from Brucella abortus induced in BALB/c mice (31).

High level of immunity induced by CD8 restricted peptides predicted for HLA-A*0201 using SYFPEITHI software was similar to those studies suggesting the computer-based prediction is more accurate for human than mouse MHC class I epitopes (21, 32), since none of the mouse MHC class I predicted peptides were immunogenic whereas the protein itself could induce CTL activity in BALB/c mouse model (unpublished data).

Immunization of HHDII mice with either C2 or CM4 peptides did not protect the mice from *L. mexicana* infection and the course of the disease in immunized mice was similar to that of the controls immunized with non-immunogenic peptide or PBS. This was in contrast with the results obtained by Spitzer in BALB/c mice using the 16-mer synthetic peptide with the sequence of YDQLVTRVVTHEMAHA derived from *L. major* gp63 (19) where the synthetic peptide protected the infected mice against the disease for 10 months. Long synthetic peptides have been recently shown to elicit anti-malaria antibody responses in human along with inhibitory effect against the parasite in vitro (23). Similar phenomena has also been demonstrated in a study on the immunogenicity of tumour antigens where 15-mer peptide containing a shorter Class I predicted epitope from HAGE protein was found to be endogenously processed and immunogenic for T cells in vivo in transgenic mice model (33). The potency of longer peptides in presenting both MHC class I and II peptides meantime and enhancing the immunity still needs to be studied. Our results may indicate that the peptide as administered is insufficient to protect mice from the infection and new approaches such as designing longer peptides containing short Class I epitopes should be considered in further studies.

## References

[1] WHO Leishmaniasis. http://www.who.int/tdr/diseases/leish/direction.htm.

[2] Griekspoor A, Sondorp E, Vos T (1999). Cost-effectiveness analysis of humanitarian relief interventions: visceral leishmaniasis treatment in the Sudan. Health Policy Plan..

[3] Desjeux P (2004). Leishmaniasis: current situation and new perspectives. Comp Immunol Microbiol Infect Dis..

[4] Kar K (1995). Serodiagnosis of leishmaniasis. Crit Rev Microbiol..

[5] Puig L, Pradinaud R (2003). *Leishmania* and HIV co-infection: dermatological manifestations. Ann Trop Med Parasitol..

[6] Sacks D, Noben-Trauth N (2002). The immunology of susceptibility and resistance to *Leishmania major* in mice. Nat Rev Immunol..

[7] Muller I, Kropf P, Etges RJ, Louis JA (1993). Gamma interferon response in secondary *Leishmania major* infection: role of CD8+ T cells. Infect Immun..

[8] Rogers KA, DeKrey GK, Mbow ML, Gillespie RD, Brodskyn CI, Titus RG (2002). Type 1 and type 2 responses to *Leishmania major*. FEMS Microbiol Lett..

[9] Erb K, Blank C, Ritter U, Bluethmann H, Moll H (1996). *Leishmania major* infection in major histocompatibility complex class II-deficient mice: CD8+ T cells do not mediate a protective immune response. Immunobiology..

[10] Awasthi A, Mathur RK, Saha B (2004). Immune response to *Leishmania* infection. Indian J Med Res..

[11] Rivier D, Bovay P, Shah R, Didisheim S, Mauël J (1999). Vaccination against *Leishmania major* in a CBA mouse model of infection: role of adjuvants and mechanism of protection. Parasite Immunol..

[12] Ali SA, Rezvan H, McArdle SE, Khodadadi A, Asteal FA, Rees RC (2009). CTL responses to *Leishmania mexicana* gp63-cDNA vaccine in a murine model. Parasite Immunol..

[13] Coler RN, Reed SG (2005). Second-generation vaccines against leishmaniasis. Trends Parasitol..

[14] Ahmad M, Rees RC, McArdle SE, Li G, Mian S, Entwisle C, Loudon P, Ali SA (2005). Regulation of CTL responses to MHC-restricted class I peptide of the gp70 tumour antigen by splenic parenchymal CD4+ T cells in mice failing immunotherapy with DISC-mGM-CSF. Int J Cancer..

[15] Assudani DP, Ahmad M, Li G, Rees RC, Ali SA (2006). Immunotherapeutic potential of DISC-HSV and OX40L in cancer. Cancer Immunol Immunother..

[16] Machado-Pinto J, Pinto J, da Costa CA, Genaro O, Marques MJ, Modabber F, Mayrink W (2002). Immunochemotherapy for cutaneous leishmaniasis: a controlled trial using killed *Leishmania* (*Leishmania*) amazonensis vaccine plus antimonial. Int J Dermatol..

[17] Stanekova Z, Vareckova E (2010). Conserved epitopes of influenza A virus inducing protective immunity and their prospects for universal vaccine development. Virol J..

[18] Fiorentini S, Marsico S, Becker PD, Iaria ML, Bruno R, Guzman CA, Caruso A (2008). Synthetic peptide AT20 coupled to KLH elicits antibodies against a conserved conformational epitope from a major functional area of the HIV-1 matrix protein p17. Vaccine..

[19] Bates PA (1994). Complete developmental cycle of *Leishmania mexicana* in axenic culture. Parasitology..

[20] Ali S, Ahmad M, Lynam J, Rees RC, Brown N (2004). Trafficking of tumor peptide-specific cytotoxic T lymphocytes into the tumor microcirculation. Int J Cancer..

[21] Saren A, Pascolo S, Stevanovic S, Dumrese T, Puolakkainen M, Sarvas M, Rammensee HG, Vuola JM (2002). Identification of *Chlamydia pneumoniae*-derived mouse CD8 epitopes. Infect Immun..

[22] Hundemer M, Schmidt S, Condomines M, Lupu A, Hose D, Moos M, Cremer F, Kleist C, Terness P, Belle S (2006). Identification of a new HLA-A2-restricted T-cell epitope within HM1.24 as immunotherapy target for multiple myeloma. Exp Hematol..

[23] Ramage JM, Metheringham R, Moss R, Spendlove I, Rees R, Durrant LG (2004). Comparison of the immune response to a self antigen after DNA immunization of HLA*A201/H-2Kb and HHD transgenic mice. Vaccine..

[24] van der Bruggen P, Bastin J, Gajewski T, Coulie PG, Boel P, De Smet C, Traversari C, Townsend A, Boon T (1994). A peptide encoded by human gene MAGE-3 and presented by HLA-A2 induces cytolytic T lymphocytes that recognize tumor cells expressing MAGE-3. Eur J Immunol..

[25] Firat H, Garcia-Pons F, Tourdot S, Pascolo S, Scardino A, Garcia Z, Michel ML, Jack RW, Jung G, Kosmatopoulos K (1999). H-2 class I knockout, HLA-A2.1-transgenic mice: a versatile animal model for preclinical evaluation of antitumor immunotherapeutic strategies. Eur J Immunol..

[26] Spitzer N, Jardim A, Lippert D, Olafson RW (1999). Long-term protection of mice against *Leishmania major* with a synthetic peptide vaccine. Vaccine..

[27] Engelhard VH, Bullock TN, Colella TA, Mullins DW (2000). Direct identification of human tumor-associated peptide antigens and a preclinical model to evaluate their use. Cancer J..

[28] Rojas JM, McArdle SE, Horton RB, Bell M, Mian S, Li G, Ali SA, Rees RC (2005). Peptide immunisation of HLA-DR-transgenic mice permits the identification of a novel HLA-DRbeta1*0101- and HLA-DRbeta1*0401-restricted epitope from p53. Cancer Immunol Immunother..

[29] Rammensee H, Bachmann J, Emmerich NP, Bachor OA, Stevanovic S (1999). SYFPEITHI: database for MHC ligands and peptide motifs. Immunogenetics..

[30] Gurunathan S, Irvine KR, Wu CY, Cohen JI, Thomas E, Prussin C, Restifo NP, Seder RA (1998). CD40 ligand/trimer DNA enhances both humoral and cellular immune responses and induces protective immunity to infectious and tumor challenge. J Immunol..

[31] Mishra S, Sinha S (2006). Prediction and molecular modeling of T-cell epitopes derived from placental alkaline phosphatase for use in cancer immunotherapy. J Biomol Struct Dyn..

[32] Pelte C, Cherepnev G, Wang Y, Schoenemann C, Volk HD, Kern F (2004). Random screening of proteins for HLA-A*0201-binding nine-amino acid peptides is not sufficient for identifying CD8 T cell epitopes recognized in the context of HLA-A*0201. J Immunol..

[33] Mathieu MG, Knights AJ, Pawelec G, Riley CL, Wernet D, Lemonnier FA, Straten PT, Mueller L, Rees RC, McArdle SE (2007). HAGE, a cancer/testis antigen with potential for melanoma immunotherapy: identification of several MHC class I/II HAGE-derived immunogenic peptides. Cancer Immunol Immunother..

